# Major Operations under Local Anesthesia—With Report of Cases

**Published:** 1915-08

**Authors:** W. A. Selman

**Affiliations:** 428 Candler Building, Atlanta, Ga.


### MAJOR OPERATIONS UNDER LOCAL ANAESTHESIA —WITH REPORT OF CASES*

By W. A. Selman, M. D.

In presenting this paper, I feel that this subject has a distinct place in major surgery, though it has for years been mostly limited to minor operations. This restricted limitation, however, was not due to any lack of surgical skill, but to the fact that until within recent years no local anaesthetic had been discovered that met the requirements of almost limitless administration with a minimum of toxicity. This problem has been most thoroughly worked out experimentally by a number of progressive surgeons foremost among whom is Dr. Crile of Cleveland. It was upon the study of his experiments and deductions that I have undertaken this work, and to him and his colleagues I gladly give credit for what knowledge I have of it.

In the selection of an anaesthetic, whether local or general, the question that should be uppermost in our minds is not under what anaesthetic can I do the best operation, but under what anaesthetic can I give my patient the most assurance of recovery. No one anaesthetic, general or local, is adapted to all cases. For instance, I regard ether as a general anaesthetic, in the vast majority of cases the one of choice. Yet in many cases

*Read before the Chattahoochee Valley Medical and Surgical Association, Opelika, Ala., July 13, 1915.

of infection ether would only add to the already heavy burden of the phagocytes and be the determining factor for a fatal termination, whereas, a gas-oxygen anaesthetic would leave the cells unharmed, and with the cause removed, win the victory.

Passing on, there is a class of cases, smaller, it is true but still sufficient to demand our attention, to which we deem it unsafe or inadvisable to give any inhalation anaesthetic. Then we have to consider spinal, intravenous, rectal or local anaesthesia, each of these methods has its advantages, its dangers, and its limitations. However, it is only with the local with which I am concerned at this time.

First, as to the local anaesthetics used: For the immediate anaesthetic effect, whether in skin, fascia, muscle or peritoneum, novocaine solution 1-400 is amply sufficient, the main precaution necessary is to inject thoroughly each layer before cutting. The novocaine alone thoroughly desensitizes the part and lasts for approximately one hour. However, other phases enter for the comfort of the patient both before and after the actual operation. As a preliminary, just as in general anaesthesia, it is usually advisable to administer a hypodermic injection of morphine, grain 1-6 and scopolamine grain 1-150, one hour before the operation. This both allays the nervousness of the patient and tends to prevent shock from the operation.

Still further to block the nerves to prevent post operative pain, a solution of quinine and urea hydrochlorid is injected into the tissues well away from the incision, yet made through the edges of the incision. This causes some oedema of the tissues, yet it is well away from the line of incision and in clean cases should not interfere with primary healing. This produces an anaesthesia that commences by the time the novocaine begins to die out, and lasts for two or three days. This quality makes post operative pain almost nil and indeed is of such marked value that many surgeons use it as a routine in conjunction with general anaesthesia for the same effect. This nerve blocking principle whether used alone or with a general anaesthetic, greatly lessens the afferent impulses to the central nervous system, consequently lessening shock from nerve stimuli just in proportion to the thoroughness with which the tissues are injected.

### REPORT OF CASES.

Case 1.—J. S., negro, age 60. Admitted to Grady Hospital March, 1914, with a strangulated right inguinal hernia, complicated with general anasarca from a cardio-renal affection so marked that the patient could not lie down, due to the pulmonary congestion, and presenting an extreme degree of oedema in all his tissues. This was a bad picture without a strangulated hernia. However, under novocaine anaesthesia, together with morphine pushed to the limit of tolerance, we succeeded in relieving the strangulation, which we found to be due to oedema of the mesentery, which instead of being a membrane had assumed a thickness of almost an inch. The intestine regained its color sufficiently to show vitality, so no resection was done. A very tedious dissection of the sac consumed a long time, as a truss had been worn for years.

A very good closure was finally gotten and he was sent to the ward with a grave prognosis. To our surprise he did better generally after the operation than he had before. He suffered no apparent shock. In fact, the morphine seemed to give all his tissues a needed rest, and he improved steadily until able to leave the hospital cured of his hernia and improved generally.

Cases 2 and 3.—These two cases being in every respect so similar, I report them together. On April 9, 1915, I was called by Dr. Burnett, of Winston, Ga., to see two children living in the same community, a boy and a girl, each seven years of age. They had pneumonia about two weeks previously. Each had an empyema involving the entire left pleural cavity.

We decided that any general anaesthetic would be dangerous. Only one lung was at work, and the patient was very septic, and drenched with perspiration. We acordingly attempted the resection of a rib for free drainage, under local anaesthesia. For this we used the ordinary hypodermic tablet of novocaine and adrenalin, making a one per cent solution of novocaine. By constantly talking to the boy concerning bird dogs, Shetland ponies, etc., we pinched up his skin and inserted a fine pointed hypodermic needle without his feeling anything but the pinch. He conversed with interest about the dogs and horses yet never realized we were operating upon him as we kept our instruments out of his view by having him turned on his side and his eyes shielded by a towel. By carefully novocainizing each layer and even the periosteum of the rib, we actually slipped a rib clip around his rib and cut out the section without a whimper from him. In a moment we had inserted a sharp pointed hemostat through the bed of the rib and had pus flowing through a rubber tube without his ever realizing that he was being operated upon.

We repeated the same technic at the next house, but the little girl scented miscief and nothing I could say got her entire attention. She did cry a little, more from fright than from pain, but soon we had her side drained in the same way, without any trouble, and when she did flinch she did it just as quickly from touching her body with the hand as from the actual cutting, for it was evidently from nervousness and fright. Both cases drained freely and improved markedly until about ten days later, the boy was seized suddenly one night with an acute attack of vomiting and died before a physician could reach him. Whether this was from an embolus or some other cause could not be ascertained.

The girl made an uneventful recovery.

Case 4.—Mr. W. C., age 30. Referred to me July 5, 1915, by Dr. Geo. M. Niles Diagnosis—chronic recurring appendicitis and active pulmonary tuberculosis.

History—About one year ago his home physician, Dr. J. W. Harper, of Jenkinsburg, Ga., diagnosed his case as pulmonary tuberculosis and treated him for it. However, each time he got him built up he would have a typical attack of appendicitis and would lose all he had gained. Examination on July 5th showed a dull area in the upper part of right lung with crepitant rales. The X-ray examination by Dr. Niles, showed decided solidification in apex of right lung and suspicious opacity in apex of left lung. Sputum showed tubercle bacilli. His abdomen was quite tender over McBurney's point, and pressure there caused pain and nausea.

He was admitted to the Georgia Baptist Hospital, and his appendix removed under novocaine anaesthesia with the preliminary hypodermic of morphine grain 1-4 and scopolamine grain 1-150 one hour before operation, with an additional 1-6 grain morphine just before operation. A McBurney gridiron operation was done, injecting each layer as it was reached. The appendix was easily brought up into the wound and was found to be quite large and uniformly thickened. Handling the appendix gently gave no acute pain but the same nauseated feeling as when deep palpation was done. A few drops of novocaine were injected into the meso appendix before ligating it, but none was used on the appendix as no pain whatever was produced when its base was grasped by a hemostat and a ligature applied. However, pulling or sponging caused him to complain some, but not acutely. His pulse never went over 94, his respiration was slow and even and he returned to his bed in comparative comfort. The quinine and urea solution prevented any acute pain, and he is now convalescing nicely, though running a little afternoon temperature from his pulmonary condition.

Case 5.—H. W., girl, 9 years, white. Referred by Drs. Bennett of Gay, Ga., and Crawford, of Locust Grove, Ga.

Admitted to the Georgia Baptist Hospital July 10, 1915, with the following history: Two weeks ago she was with other children watching a tennis game. A stray bullet shot at a dog 150 yards away passed through her left arm entering the left side of chest in axilla, passed out of right side of chest just posterior to mid axiliary line and at the level of the sixth intercostal space. The bullet lodged in her clothing and fell at her feet, unscratched by any bone or other solid object. She was put to bed and her left pleural cavity soon filled with fluid so completely that dullness extended from base to apex with no transmission of sounds.

On day of operation at 12:30 P. M., she was given 1-300 grain of scopolamine and 1-12 grain of morphine. At 1:30 under novocaine a section of her rib was resected with no pain until the rib clip was passed around the rib, and then the patient only complained a little and did not even cry. The pleura was then opened and the fluid found to be blood; thin, dark, and apparantly disintegrating clots.

Her temperature was 102 2-5 at time of operation. It went to 103 that night at 10 o'clock then gradually dropped to 99 and has remained at about that since. She has been in comparative comfort and is now convalescing.

In conclusion, though my cases are few, I am convinced that many cases of pulmonary, cardiac, renal or other systemic affections that would make dangerous a general anaesthetic, can yet in many instances be safely relieved of surgical conditions that make existence miserable. Many people are trudging along through life with a constantly nagging rectal condition or with a hernia that slips under a truss with every strain. Possibly some one has told him never to take an anaesthetic. To those who fear an anesthetic from some fancied trouble, and to those who have real cause to fear a general anaesthetic, local anaesthesia should be welcomed as a friend indeed.

428 Candler Building, Atlanta, Ga.

